# I-OPen: inferior outcomes of penta-refractory compared to penta-exposed multiple myeloma patients

**DOI:** 10.1038/s41408-022-00733-2

**Published:** 2022-09-23

**Authors:** Sarvarinder K. Gill, Rashmi Unawane, Shuqi Wang, Jaeil Ahn, Adolfo Aleman, David S. Siegel, David H. Vesole, Harsh Parmar, Pooja Phull, Noa Biran

**Affiliations:** 1 Department of Medicine, Hackensack Meridian School of Medicine, Hackensack University Medical Center, Hackensack, NJ USA; 2 Division of Multiple Myeloma, John Theurer Cancer Center, Hackensack Meridian School of Medicine, Hackensack University Medical Center, Hackensack, NJ USA; 3 Department of Biostatistics, Bioinformatics, and Biomathematics, Georgetown University, Washington, DC USA

**Keywords:** Myeloma, Translational research

## To the Editor,

Multiple myeloma (MM) is a clinically and biologically heterogeneous malignancy characterized by structural and numerical chromosomal abnormalities, mutational and copy number abnormalities that have impact on prognosis and responsiveness to various therapies [1, 2]. The treatment paradigms and outcomes for patients with MM have improved significantly over the past 15 years with increased understanding of the disease biology and expansion of therapeutic options [3, 4]. The five most active anti MM drugs that define the term “penta-refractory” are proteasome inhibitors (PIs) bortezomib and carfilzomib, immunomodulatory drugs (IMiDs) lenalidomide and pomalidomide and the anti-CD38 monoclonal antibodies (CD38 MoABs) [4, 5]. Triplet and quadruplet therapies for induction and/or relapse have led to improved survival [6, 7]. Despite these advances in treatments, relapse of MM is inevitable. With each relapse, there may be acquisition of new mutations, epigenetic changes, and changes in the bone marrow microenvironment but there is also shift in the distribution of preexisting clones as selective pressures are applied rendering the disease more resistant and leading to ultimate development of relapsed/refractory MM (RRMM), extramedullary disease, and plasma cell leukemia, where further options are unlikely to result in deep or durable remissions [8, 9].

To date, there have been no large-scale single-center “real-world” studies with long-term follow-up among quad-and penta exposed and/or refractory MM patients. We report the patient characteristics of this patient population to provide a benchmark for new therapies.

The study included consecutive patients from John Theurer Cancer Center at Hackensack University Medical Center who were quad and penta exposed and/or refractory between the dates of 1/1/2015 and 3/1/2021. Quad-exposed was defined as having had prior exposure to two proteasome inhibitors (PIs): bortezomib or ixazomib and carfilzomib and 2 immunomodulatory drugs (IMiDs): lenalidomide and pomalidomide. Penta-exposed was defined as having prior exposure to 2 PIs and 2 IMiDs and additional exposure to an anti-CD38 monoclonal antibody (CD38 MoABs). Penta or quad refractory was defined as having stable disease (as best response) or progressive disease while on all of the above drugs, per International Myeloma Working Group (IMWG) definition of refractory [10]. The time point at which patients met the above criteria for progression was referred to as time zero (T0^r^) for the refractory group and the time point of exposure to the last drug of the above treatment was referred to as T0^e^ for the quad/penta exposed group.

Study data were collected between March 2021 and November 2021 and managed using electronic data tools available at Hackensack University Medical Center. For analysis, patients were classified into four groups based on Quad/Penta and Exposed/Refractory: Quad exposed, Quad refractory, Penta exposed and Penta refractory. Overall response rate (ORR) used the IMWG criteria. Overall survival (OS) time was measured from T0 until death or last follow up. For the subset of patients who underwent further therapy after T0, we analyzed the response to subsequent therapy and progression-free survival (PFS), here defined as the time between the onset of the next line of therapy and disease progression or death.

We compared categorical variables between two or more groups utilizing Fisher’s exact test or Pearson’s chi-squared test and continuous variables utilizing the Kruskal-Wallis non-parametric test. For ORR, the exact binomial 95% confidence interval (CI) was calculated. The median PFS and OS were estimated using the Kaplan-Meier method for the entire population and for subgroups of patients. Comparisons of OS and PFS between groups were performed using a log-rank test. Univariate and multivariable adjusted Cox proportional hazard regression models examined patient-disease and treatment features affecting OS.

One hundred and sixty patients were included in this study: the median age at diagnosis was 68.5 (range 60–74), 56% patients were male and 44% were stratified as high risk. Extramedullary disease was present in 24% of patients. The majority of patients (*N* = 109, 68%) were “penta-refractory”, 32 (20%) were “quad - refractory” and 19(12%) were “quad and penta exposed”. Median interval from diagnosis of MM to T0 was 59 months (IQR 37–99) for all patients. The median number of prior therapies for all patients was 6 (IQR 4–8) prior to T0; 82% of all patients underwent prior autologous hematopoietic cell transplantation (AHCT).

**Fig. 1:**
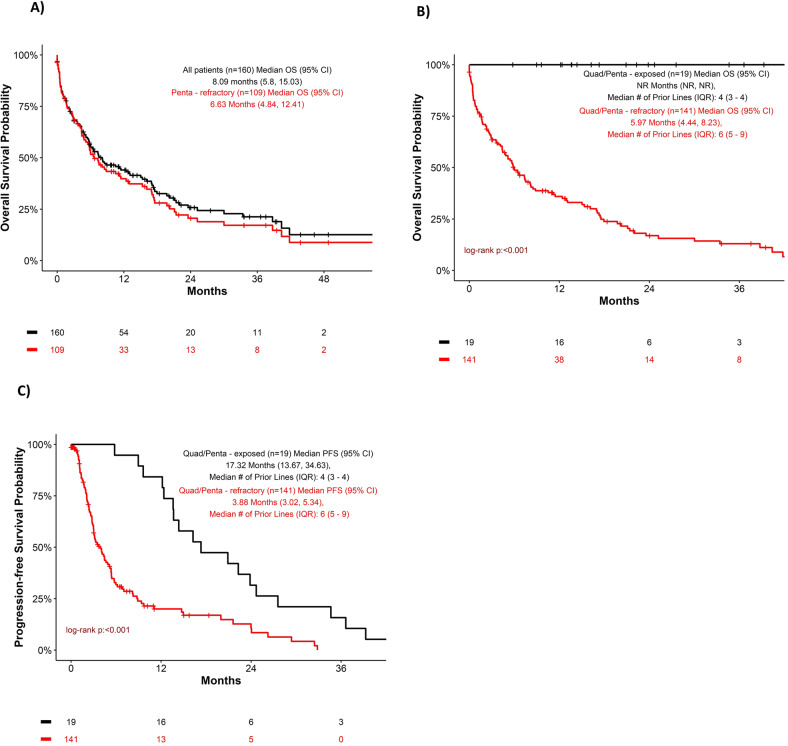
Kaplan Meier curve. Overall OS (**A**), OS for quad/penta exposed versus quad/penta refractory (**B**), and PFS for quad/penta exposed versus quad penta refractory (**C**) OS = Overall survival; PFS = progression free survival.

The median overall survival (OS) from the time of penta or quad refractoriness or exposure (T0) for the entire cohort was 8.09 months (95% CI, 5.8–15.03). The median OS for the penta- refractory group was 6.6 months (95% CI, 4.8–12.4) (Fig. 1a). The median OS for the quad/penta refractory group combined was 6.0 months (95% CI, 4.4–8.2) versus median OS for quad/penta exposed group was not reached (NR) (Fig. 1b). The median progression free survival (PFS) for Quad/Penta refractory groups combined was 3.88 months (95% CI, 3.02–5.34) vs median PFS of Quad/Penta exposed groups 17.3 months (95% CI, 13.7–34.6) (Fig. 1c).

On univariate and multivariate analyses, no prior treatment impacted OS. For the subgroup of penta-refractory patients who were quad and penta-refractory non-simultaneously, those who had ≤10 months between becoming quad- and penta-refractory had inferior OS compared to patients with >10 months using multivariate analysis (*p* < 0.01).

The cohort of 111 patients who had received at least one line of therapy post-T0 were categorized according to the agent or combination of agents used in the first subsequent line of therapy to report key characteristics and therapeutic outcomes (Table 1). The ORR was 20% with PFS and OS of 2.7 months (95% CI, 2.1–3.4) and 8.1 months (95% CI, 5.8–15.0) respectively.

**Table 1:** Patient characteristics and outcomes of specific regimens at next line after T0.

Patient characteristics											
	All Regimens *N* = 111	Any dara^d^ *N* = 23	Dara + IMiD *N* = 14	Dara + PI *N* = 12	Any carfil *N* = 46	Carfil + IMiD *N* = 20	Carfil + Other^c^ *N* = 2	Selinexor *N* = 7	Bendamustine *N* = 14	CAR-T *N* = 4	Any dara vs any carfil pvalue
High-risk FISH	40 (36.0%)	8 (34.8%)	4 (28.6%)	4 (33.3%)	19 (41.3%)	10 (50.0%)	0 (0.0%)	3 (42.9%)	5 (35.7%)	2 (50%)	0.8377
Prior Lines^a^	7 (3–22)	8 (3–22)	7.5 (3–15)	8 (3–22)	7 (3–22)	7 (3–22)	7.5 (6–9)	6 (4–9)	7 (4–10)	7.5 (6–8)	0.3430
Penta refractory	91 (82.0%)	23 (100.0%)	14 (100.0%)	12 (100.0%)	34 (73.9%)	16 (80.0%)	1 (50.0%)	7 (100.0%)	7 (50.0%)	4 (100.0%)	--
Quad refractory	20 (18.0%)	0 (0.0%)	0 (0.0%)	0 (0.0%)	12 (26.1%)	4 (20.0%)	1 (50.0%)	0 (0.0%)	7 (50.0%)	0 (0.0%)	--
Outcomes of next line regimen after T0 for quad and penta refractory patients											
PD	55 (49.6%)	11 (47.8%)	7 (50.0%)	6 (50.0%)	21 (45.6%)	10 (50.0%)	0 (0.0%)	3 (42.8%)	11 (78.6%)	0 (0.0%)	
SD	34 (30.6%)	7 (30.4%)	3 (21.4%)	3 (25.0%)	16 (34.8%)	5 (25.0%)	2 (100.0%)	3 (42.9%)	3 (21.4%)	0 (0.0%)	
PR	10 (9.0%)	4 (17.4%)	3 (21.5%)	3 (25.0%)	5 (10.9%)	4 (20.0%)	0 (0.0%)	0 (0.0%)	0 (0.0%)	1 (25.0%)	
VGPR	6 (5.4%)	0 (0.0%)	0 (0.0%)	0 (0.0%)	3 (6.5%)	1 (5.0%)	0 (0.0%)	1 (14.3%)	0 (0.0%)	0 (0.0%)	
CR/sCR	6 (5.4%)	1 (4.3%)	1 (7.1%)	0 (0.0%)	1 (2.2%)	0 (0.0%)	0 (0.0%)	0 (0.0%)	0 (0.0%)	3 (75.0%)	
ORR^b^	22 (19.8%)	5 (21.7%)	4 (28.6%)	3 (25.0%)	9 (19.6%)	5 (25.0%)	0 (0.0%)	1 (14.3%)	0 (0.0%)	4 (100.0%)	1.0000
PFS and OS (in months (95% C.I.)) on next line after T0											
PFS	2.7 (2.1, 3.4)	2.8 (1.5, 4.9)	2.9 (1.3, 12.1)	2.6 (1.8, NR)	2.5 (2.1, 3.8)	2.7 (2.0, 4.9)	4.4 (3.8, NR)	4.5 (1.8, NR)	2.0 (1.3, 3.4)	18.7 (7.8, NR)	1.0000
OS	8.1 (5.8, 15.0)	7.4 (5.6, 23.6)	5.7 (0.4, NR)	8.8 (6.0, NR)	5.8 (4.4, 15.0)	5.0 (1.0, 17.5)	12.7 (5.1, NR)	NR (NR, NR)	6.4 (4.4, NR)	NR (10.5, NR)	0.3000

*NR* not reached, *T0* time at which patient becomes Quad/Penta refractory.

^a^Median (Range).

^b^Overall response rate = proportion of getting partial response or better.

^c^Panobinostat or venetoclax.

^d^3 patients have Dara + IMiD +PI.

A carfilzomib-based (*N* = 68) regimen was most used as the first subsequent lines of therapy post-T0. The categorization of groups was not mutually exclusive. All patients were refractory to 2 IMiDs and 2 PIs. There was no statistically significant difference in median PFS and median OS (*p* value 1.0 and 0.3 respectively) with using any daratumumab vs any carfilzomib based regimen as next line therapy after T0. Patients treated with CAR-T as first line after T0 has PFS of 18.7 and OS was not reached.

Our results of this “real-world” single institution retrospective study of quad/penta exposed or refractory multiple myeloma patients demonstrates their overall dismal outcomes, thus defining the unmet need in this population. The median PFS for the quad/penta refractory patients was 3.9 months and the median OS was 6 months. Those who were refractory versus exposed had a median PFS of 3.9 months versus 17 months respectively (*p* < 0.05). Those who were refractory had a median OS of 6 months versus OS not reached for the exposed group.

Results from this study are consistent with those from similar longitudinal studies of patients with RRMM [11–14]. The median PFS of 3.9 months in our quad/penta refractory patients is similar to the median PFS of 3.0 in triple class refractory patients in the LocoMMotin study that assessed the effectiveness of real-life standard of care treatments in triple-class exposed patients. Our median OS of 8 months for the entire cohort is similar to median OS survival of 8.6 months seen in the retrospective MAMMOTH study that investigated the outcomes of patients refractory to CD38 MoAB. Median OS of 6.6 months in our penta-refractory patients is similar to the 5.6 months seen in the MAMMOTH study. Although difficult to compare across studies, when comparing the quad/penta exposed patients in this study versus the non-triple refractory patients of the prospective LocoMMotion study and the non-triple refractory patients from the MAMMOTH study, we see significantly improved median PFS 17 months versus 8.2 months (LocoMMotion) versus 9.2 months (MAMMOTH). This emphasizes that once patients become quad/ penta refractory, there is a significant decrease in effectiveness of therapies with shorter PFS and low OS.

Our data suggests that specific sequencing of therapies earlier in the disease course does not make a difference once the patients develop RRMM. Carfilzomib-based regimens were the most-widely employed first subsequent treatment after T0 in our study. Patients who received carfilzomib-based therapies as their first line after T0 had PFS of 2.5 months with ORR of 20% similar to daratumumab-based regimens which had PFS of 2.8 months with ORR of 22%. This highlights that after patients have become quad/penta refractory, no one standard regimen is more efficacious given they had been exposed to those agents previously. Some patients were treated with CAR-T therapy later as their second or third line after T0. Patients treated with CAR-T as first line after T0 had PFS of 18.7 and OS was not reached. This emphasizes that CAR T-cell therapy may be highly effective for patients who have RRMM not responded to multiple prior treatments.

This study serves as a large database of patients, heterogeneously treated, and heavy refractory.

In summary, this study demonstrates the poor prognosis of penta/quad refractory patients, and the unmet need for novel therapies that this patient population represents. There is no established standard of care treatment or sequencing of therapies for penta or quad RRMM patients. Recent approval of cellular therapies and perhaps the forthcoming bispecific T-cell engagers will represent a significant milestone in treatment of RRMM [15].

## Supplementary information

Supplementary Tables

## Data Availability

The datasets generated during and/or analyses during the current study are available from the corresponding author on reasonable request.
