# Supplementary material for: I-OPen: inferior outcomes of penta-refractory compared to penta-exposed multiple myeloma patients
Source: Blood Cancer J. 2022 Sep 23;12(9):138. doi: 10.1038/s41408-022-00733-2 (PMC9508328; doi:10.1038/s41408-022-00733-2)
Supplement: Supplementary file 1 — Supplementary Tables [file 41408_2022_733_MOESM1_ESM.docx]

**Table 1:** Baseline Characteristic

| **Characteristics** | | **All**  (n=160) | **Quad/Penta - exposed**  (n=19) | **Quad - refractory**  (n=32) | **Penta - refractory**  (n=109) | **P - value** |
| --- | --- | --- | --- | --- | --- | --- |
| Age at T0*  (median (IQR)) | | 68.50  (60.75 - 74.00) | 68.00  (62.00 - 71.00) | 70.00  (56.00 - 73.5) | 69.00  (61.00 - 75.00) | 0.6417 |
| Gender Male (%) | | 89 (56.0) | 11 (57.9) | 16 (50.0) | 62 (57.4) | 0.7475 |
| Ig Subtype (%) | |  |  |  |  | 0.1655 |
|  | IgA | 35 (21.9) | 1 (5.3) | 7 (21.9) | 27 (24.8) |  |
|  | IgG | 96 (60.0) | 16 (84.2) | 20 (62.5) | 60 (55.0) |  |
|  | LC | 4 (2.5) | 1 (5.3) | 1 (3.1) | 2 (1.8) |  |
|  | Other** | 25 (15.6) | 1 (5.3) | 4 (12.5) | 20 (18.3) |  |
| ISS at Diagnosis (%) | |  |  |  |  | 0.3028 |
|  | 1 | 49 (30.6) | 6 (31.6) | 10 (31.2) | 33 (30.3) |  |
|  | 2 | 66 (41.2) | 8 (42.1) | 18 (56.2) | 40 (36.7) |  |
|  | 3 | 39 (24.4) | 4 (21.1) | 4 (12.5) | 31 (28.4) |  |
|  | Unknown^†^ | 6 (3.8) | 1 (5.3) | 0 (0.0) | 5 (4.6) |  |
| Stratification risk High (%) | | 70 (43.8) | 11 (57.9) | 10 (31.2) | 49 (45.0) | 0.1620 |
| Number of lines  (median (IQR)) | | 6.00  (4.00 - 8.00) | 4.00  (3.00 - 4.00) | 5.50  (4.75 - 7.00) | 7.00  (5.00 - 9.00) | <0.0001 |
| Prior ASCT Yes (%) | | 131 (81.9) | 17 (89.5) | 24 (75.0) | 90 (82.6) | 0.4577 |
| Extramedullary Disease Yes (%) | | 39 (24.8) | 4 (21.1) | 6 (19.4) | 29 (27.1) | 0.6890 |
| Plasma Cell Leukemia Yes (%) | | 14 (8.9) | 0 (0.0) | 3 (9.7) | 11 (10.2) | 0.4280 |
| Creatinine at diagnosis  (median (IQR)) | | 0.99  (0.8 - 1.4) | 0.90  (0.80 - 1.40) | 0.95  (0.80 - 1.10) | 1.04  (0.82 - 1.48) | 0.3890 |
| LDH at diagnosis (median (IQR)) | | 212 .0  (161.0 - 460.0) | 189.0  (147.3 - 248.3) | 291.5  (188.5 - 495.5) | 198.0  (161.0 - 475.0) | 0.2329 |
| Drugs at 1st relapse (%) | |  |  |  |  | 0.0512 |
|  | Carfil based regimen | 28 (17.5) | 4 (21.1) | 5 (15.6) | 19 (17.4) |  |
|  | Dara based regimen | 21 (13.1) | 6 (31.6) | 0 (0.0) | 15 (13.8) |  |
|  | IMID based doublet (rev/dex or pom/dex) | 54 (33.8) | 4 (21.1) | 13 (40.6) | 37 (33.9) |  |
|  | Bortezomib based triplet | 40 (25.0) | 2 (10.5) | 11 (34.4) | 27 (24.8) |  |
|  | Other | 17 (10.6) | 3 (15.8) | 3 (9.4) | 11 (10.1) |  |
| Time from Quad to Penta -refractory^#^ (median (IQR)), months | | N/A | N/A | N/A | 10.00  (3.71 - 16.44) | N/A |
| Time from diagnosis of MM to T0 (median (IQR)), months | | 59.38  (37.23 – 98.94) | 52.21  (39.82 – 77.32) | 46.04  (30.82 - 85.10) | 67.34  (40.87 - 104.32) | 0.0747 |

Note: (1) *: T0 - time at which patient becomes Quad/Penta exposed/refractory

(2) **: Other Ig subtype includes IgD, non-secretory, oligosecretory and unspecified

(3) ^#^: Only for patients who did not become Quad – refractory and Penta – refractory at the same day, n=52

(4) ^†^: Unknown is not included in statistical test.

**Table 2:** Univariate and Multivariate Analysis for Factors affecting OS for all patients. Variables are selected if p<0.2 in univariate analysis

|  | | Univariate (n=160) | | Multivariate (n=119) | |
| --- | --- | --- | --- | --- | --- |
|  |  | HR (95% CI.) | P value | HR (95% CI.) | P value |
| Stratification Risk | |  |  |  |  |
|  | Standard | 1.00 (reference) |  |  |  |
|  | High | 1.17 (0.80, 1.71) | 0.4150 | -- | -- |
| Extramedullary Disease | |  |  |  |  |
|  | No | 1.00 (reference) |  |  |  |
|  | Yes | 0.95 (0.62, 1.46) | 0.8170 | -- | -- |
| Creatinine at diagnosis | |  |  |  |  |
|  | <1.5 | 1.00 (reference) |  |  |  |
|  | ≥1.5 | 1.27 (0.78, 2.06) | 0.3300 | -- | -- |
| LDH at diagnosis | |  |  |  |  |
|  | <200 | 1.00 (reference) |  | 1.00 (reference) |  |
|  | ≥200 | 1.44 (0.91, 2.25) | 0.1160 | 1.53 (0.95, 2.47) | 0.0818 |
| Drug 1st relapse | |  |  |  |  |
|  | Carfil based regimen | 1.00 (reference) |  | 1.00 (reference) |  |
|  | Dara based regimen | 0.47 (0.22, 1.01) | 0.0520 | 0.56 (0.24,1.30) | 0.1783 |
|  | IMID based doublet (rev/dex or pom/dex) | 0.67 (0.39, 1.17) | 0.1580 | 0.65 (0.34, 1.27) | 0.2086 |
|  | Bortezomib based triplet | 0.76 (0.43, 1.36) | 0.3620 | 0.84 (0.42, 1.70) | 0.6355 |
|  | Other | 0.61 (0.30, 1.26) | 0.1840 | 0.46 (0.17, 1.24) | 0.1231 |

*T0 - time at which patient becomes Quad/Penta exposed/refractory

**number of missing: Extramedullary Disease (n=3), Creatinine at diagnosis (n=19), LDH at diagnosis (n=41)

**Table 3:** Univariate and Multivariate Analysis for Factors affecting OS for penta-refractory patients with time from Quad to Penta-refractory >0 only. Variables are selected if p<0.2 in univariate analysis

|  | | Univariate (n=52) | | Multivariate (n=52) | |
| --- | --- | --- | --- | --- | --- |
|  |  | HR (95% CI.) | P value | HR (95% CI.) | P value |
| Stratification Risk | |  |  |  |  |
|  | Standard | 1.00 (reference) |  |  |  |
|  | High | 1.45 (0.75, 2.78) | 0.2680 | -- | -- |
| Extramedullary Disease | |  |  |  |  |
|  | No | 1.00 (reference) |  |  |  |
|  | Yes | 1.00 (0.49, 2.05) | 0.9910 | -- | -- |
| Creatinine at diagnosis | |  |  |  |  |
|  | <1.5 | 1.00 (reference) |  |  |  |
|  | ≥1.5 | 1.26 (0.56, 2.82) | 0.5780 | -- | -- |
| LDH at diagnosis | |  |  |  |  |
|  | <200 | 1.00 (reference) |  |  |  |
|  | ≥200 | 1.12 (0.52, 2.40) | 0.7650 | -- | -- |
| Drug 1st relapse | |  |  |  |  |
|  | Carfil based regimen | 1.00 (reference) |  | 1.00 (reference) |  |
|  | IMID based doublet (rev/dex or pom/dex) | 0.73 (0.30,1.75) | 0.4760 | 0.95 (0.38, 2.34) | 0.9060 |
|  | Bortezomib based triplet | 0.61 (0.25, 1.48) | 0.2710 | 0.73 (0.30,1.80) | 0.4983 |
|  | Other | 0.40 (0.10, 1.56) | 0.1880 | 0.46 (0.12, 1.82) | 0.2690 |
| Time from Quad to Penta - refractory | |  |  |  |  |
|  | $>10$ months | 1.00 (reference) |  |  |  |
|  | $\leq10$ months | 2.27 (1.18, 4.39) | 0.0144 | 2.28 (1.16, 4.49) | 0.0171 |

*T0 - time at which patient becomes Penta refractory

**number of missing: Extramedullary Disease (n=2), Creatinine at diagnosis (n=7), LDH at diagnosis (n=12)

***Median number of prior lines for Penta - refractory patients with time from Quad to Penta-refractory >0 only is 8 (IQR: 6.75 - 10). Among these patients, it is 9.5 (IQR: 8.25 - 10.75) for patients with time from Quad to Penta – refractory >10 months and 7 (IQR: 5 - 8) for patients with from Quad to Penta – refractory ≤10 months.
